# Successful Co-administration of Dupilumab and Anti-tuberculosis Drugs in a Severely Uncontrolled Asthma Patient With Pulmonary Tuberculosis

**DOI:** 10.7759/cureus.66807

**Published:** 2024-08-13

**Authors:** Aadil Ashraf Ahmed Shaikh, Mary Ann Boniface, Syed Ammar Husain, Nida Naeem, Syed Arshad Husain

**Affiliations:** 1 Department of Pulmonary Medicine, Kings College Hospital London, Dubai, ARE; 2 Pulmonology Department, Brighton and Sussex National Health Service (NHS) Trust, Brighton, GBR

**Keywords:** case report, biologics, asthma, tuberculosis, dupilumab

## Abstract

The co-occurrence of active tuberculosis (TB) in patients with moderate to severe asthma presents unique therapeutic challenges, particularly with the advent of biologics like dupilumab, which targets the interleukin-4 (IL-4) and interleukin-13 (IL-13) pathways in asthma treatment. Despite the general safety of biologics, concerns about immunosuppression and susceptibility to infections like TB persist. This case report discusses a male with severe, uncontrolled type 2 allergic asthma, who experienced multiple exacerbations despite maximal bronchodilator therapy and then concomitantly developed pulmonary TB. This case demonstrates a potential clinical scenario for co-administering dupilumab with anti-TB treatment, suggesting a beneficial approach for similar clinical scenarios and contributing to the literature on this topic.

## Introduction

Tuberculosis (TB), an infectious disease caused by *Mycobacterium tuberculosis*, primarily affects the pulmonary system but can also spread to other organs. It is the leading cause of death from infectious diseases worldwide. Biologics is a class of medications that modulate the immune system to reduce inflammation. It has significantly advanced the treatment of various inflammatory conditions since the introduction of the first biologic drug in 1980. Among the five biologic medications licensed under the National Institute for Health and Care Excellence (NICE)for managing severe asthma, dupilumab (Dupixent®) is licensed as an add-on maintenance therapy [1,2]. It is indicated for patients with severe asthma with type 2 inflammation that remains inadequately controlled on maximal bronchodilator therapy, including the requirement of high-dose inhaled corticosteroids. We present our experience in managing a patient with severe uncontrolled asthma and concomitant occurrence of pulmonary TB.

## Case presentation

We present the case of a 39-year-old male from South Africa with a longstanding history of severe, uncontrolled type 2 allergic asthma. Despite being on maximal asthma bronchodilator therapy, including long-acting beta-agonists (LABA), inhaled corticosteroids (ICS), tiotropium inhalers, and leukotriene receptor inhibitor montelukast, the patient had experienced multiple exacerbations necessitating the use of oral corticosteroids*. *

Case history

**Figure 1:**
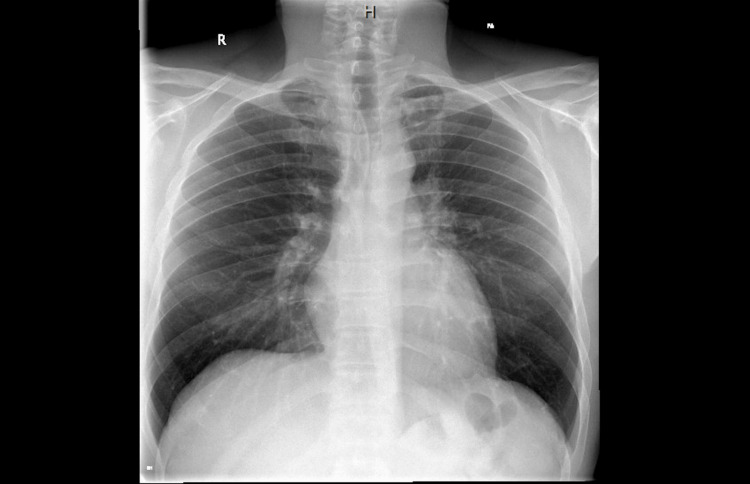
Initial X-ray of the chest Taken on April 14, 2022.

The patient presented to our clinic in April 2022 with severe cough, chest tightness, shortness of breath (SOB), and wheezing, particularly on exertion. Physical examination revealed wheezing and stridor. Initial investigations revealed elevated levels in both exhaled nitric oxide (FeNO) and immunoglobulin (IgE) tests, with IgE measuring 1079 and FeNO at 42 parts per billion (PPB). In conjunction, the chest X-ray obtained on April 14, 2022, was clear of any abnormalities (Figure 1).

The patient was prescribed beclomethasone-formoterol and advised to follow up. However, he missed subsequent appointments and returned in June 2022 with a severe exacerbation. Montelukast and tiotropium were added to his regimen. Despite these interventions, his symptoms remained poorly controlled through July and August 2022. He experienced persistent productive cough, wheezing, and SOB. Due to the severity of his symptoms, he was prescribed oral steroids (40 mg) in August 2022, which initially improved his condition. However, he relapsed within two weeks of tapering down the steroids, experiencing another severe exacerbation. Given his profile of uncontrolled type 2 asthma and declining quality of life, initiation of biologic therapy with dupilumab was considered in October 2022.

Diagnostics

**Figure 2:**
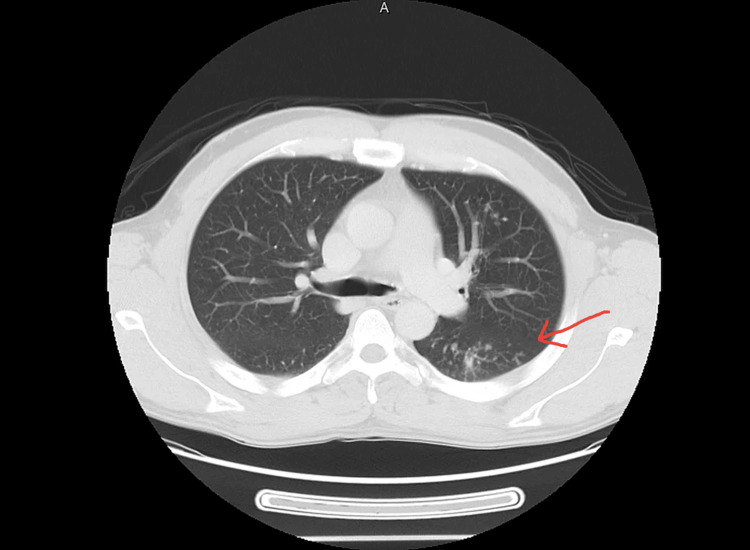
CT scan of the chest Performed in October 2022.

Computed Tomography (CT) scan of the chest (Figure 2) in October 2022 revealed reticulonodular opacities and ground glass nodules of varying sizes in the left upper zone, along with sub-centimeter mediastinal and hilar lymphadenopathy, suggesting an infectious etiology. Bronchoscopy with bronchoalveolar lavage (BAL) later confirmed the presence of acid-fast bacilli and mixed growth of gram-negative rods and gram-positive cocci. The patient was symptomatic with a fever of 38.9°C, prompting isolation and treatment discussions. His HIV status was found to be negative, and close contact tracing was completed.

Treatment Plan

Ciprofloxacin was initiated to cover gram-negative rods and gram-positive cocci. Considering his ongoing allergic type 2 asthma, which remained uncontrolled despite maximum therapy, biologic therapy with dupilumab was considered. Due to uncertainty about coadministering dupilumab and anti-TB therapy, we reviewed the literature and consulted the education and research department of dupilumab's manufacturer, Sanofi, before starting the patient on both treatments.

Dupilumab was started on November 9, 2022, less than two weeks before the initiation of anti-tuberculosis therapy which was initiated on November 21, 2022. The anti-tuberculosis regimen, RIPE therapy (rifampicin, isoniazid, pyrazinamide, ethambutol), was commenced for a scheduled duration of six months. An ophthalmologic examination was conducted before starting ethambutol, and the patient was advised to avoid close contact with his partner for one month.

**Figure 3:**
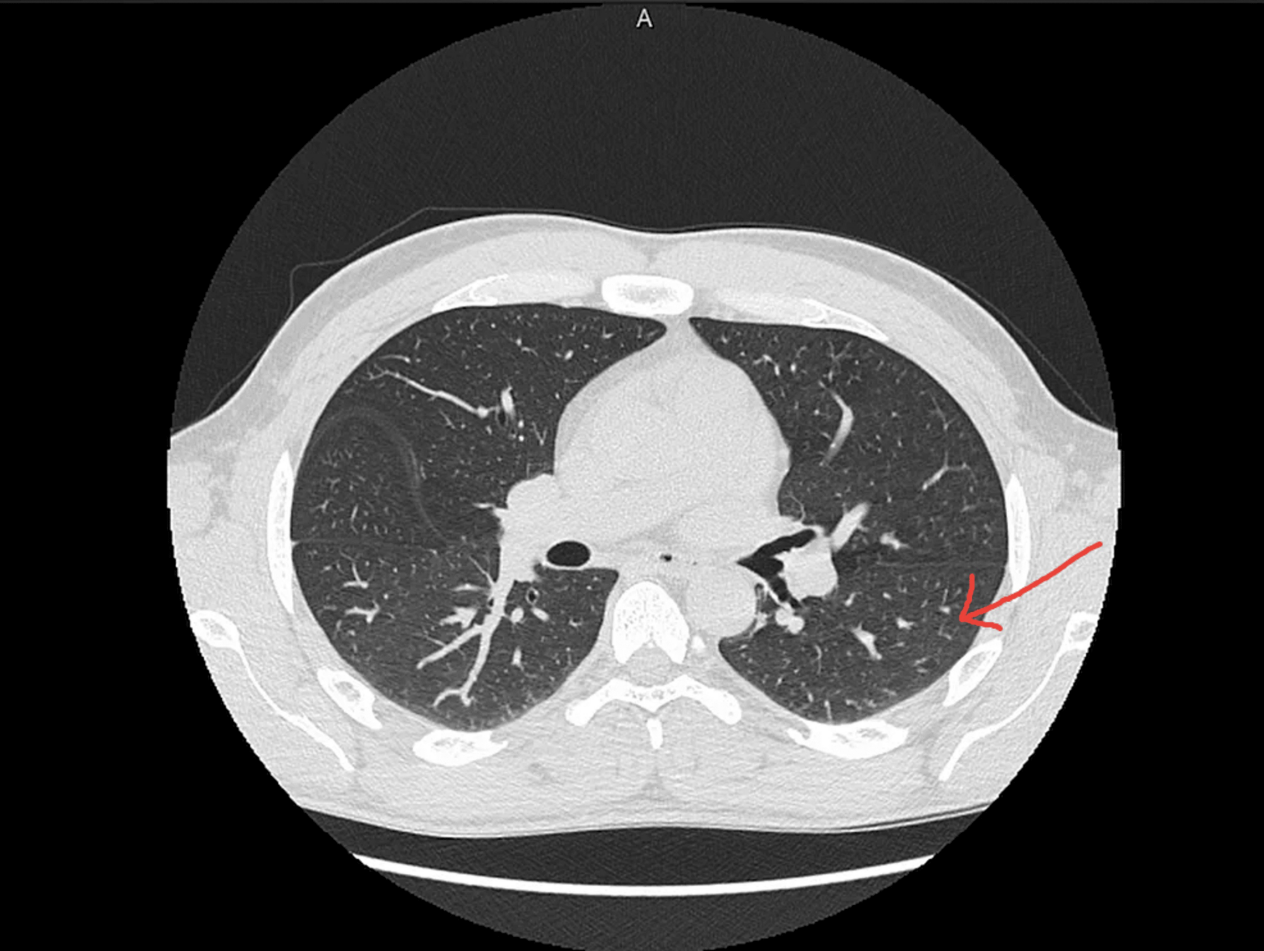
Follow-up CT scan of the chest Done on August 11, 2023.

The patient continued to receive dupilumab injections while undergoing the anti-TB drug regimen. He demonstrated considerable improvement in asthma control, reporting only mild wheeziness and occasional cough, a significant improvement from his previous condition. During the treatment period, he had regular follow-up visits with a pulmonologist and was found to be progressing well. Despite the initial social withdrawal due to his overlying condition, he began exercising and felt better overall once commencing treatment. After two months of treatment, the patient reported an improvement in asthma symptoms, evidenced by a decrease in FeNO from 46 PPB in April 2022 to 26 PPB in January 2023. After five months of anti-TB therapy, the patient reported significant improvement, started going to the gym, and actively participated in sports, describing a transformative improvement in his quality of life. Figure 3 below shows a follow up chest CT scan which was taken 10 months after the initial CT, this CT scan shows significant improvement of TB and mild bronchiolar dilatation.

Asthma control was regularly assessed using Asthma Control Tests (ACTs). His initial ACT score on November 9, 2022, was 7, which improved to 19 in subsequent assessments. He successfully completed anti-tuberculosis treatment in April 2023. Although he reported eye pain, evaluations by both a neurologist and ophthalmologist did not determine the cause, and symptoms improved with ibuprofen. Six months post-treatment, he reported good asthma control with an ACT score of 24 and could exercise and participate in activities without difficulty. More than a year after completing TB treatment, he is continuing to take dupilumab twice a month and is able to administer it to himself at home. One year later, his ACT score was 25, indicating long-term symptom control.

## Discussion

In recent years, the use of biologics has significantly advanced the management of moderate to severe type 2 asthma. Dupilumab, a human monoclonal antibody targeting the alpha subunit of the interleukin-4 receptor, effectively inhibits IL-4 and IL-13 signaling, which are crucial pathways in type 2 inflammatory diseases such as asthma [3]. A systematic review in 2022 analyzing data from 12 recent randomized controlled trials demonstrated improvement in key clinical parameters, including forced expiratory volume in 1 second (FEV1), asthma control questionnaire (ACQ) scores, fractional exhaled nitric oxide (FENO) levels, and immunoglobulin E (IgE) levels at both 12 and 24 weeks of treatment [4]. Dupilumab was generally well-tolerated, with no significant difference in adverse events compared to placebo. Consequently, dupilumab decreases the need for high-dose oral corticosteroids and improves quality of life by reducing the incidence of adverse effects associated with corticosteroids, such as Cushing syndrome and osteoporosis.

In this case report, we describe the successful co-administration of dupilumab with an anti-tuberculosis medication regimen in a patient with severely uncontrolled asthma and active TB. This approach to treatment has not been widely documented, and our aim was to report any adverse effects and treatment outcomes. It is important to note that there is limited literature on the effect of biologics like dupilumab on active TB infection or in patients concomitantly taking anti-tuberculosis medications.

For instance, Siji et al. reported a case where a patient with refractory pemphigus vulgaris and pulmonary TB was effectively managed with dupilumab as an add-on therapy [4]. This case suggests that dupilumab has been used safely in a patient with pemphigus vulgaris complicated by TB. Furthermore, a study by Yuan et al. indicated that using prophylaxis therapy (anti-TB therapy) while administering biologic treatments (adalimumab, secukinumab, guselkumab) for psoriatic patients with latent TB infection significantly reduced TB reactivation by 14% and decreased adverse events associated with biologic treatment [5]. To our knowledge, our case is unique as it is the first reported instance of using dupilumab for bronchial asthma with concomitant pulmonary TB.

Cytokine-driven cellular immunological pathways play a key role in the clearance of *Mycobacterium tuberculosis *(MTB) infection. Cytokines such as IL-4 are particularly crucial in altering the balance between Th1 and Th2 responses. Higher levels of IL-4 have been discovered in individuals with advanced stages of TB, potentially negatively impacting TB patients. However, studies like those by Shah et al. report that while the expression of IL-13 and IL-4R may play a role in TB reactivation, they are not essential in fighting TB infection [6]. Dupilumab is thought to have a low risk of causing TB infection and may even be protective against TB reactivation due to its ability to inhibit this ligand-receptor interaction. However, there is limited data to support this theory [6,7].

We have previously used Dupilumab successfully in patients with moderate to severe uncontrolled asthma, finding it to be positively life-changing. This rationale led us to prescribe it to our patient with severe asthma. Our patient, treated with both dupilumab and an anti-TB RIPE regimen, was followed and found to have a positive outcome with no drug interactions or any adverse effects. Lung improvement was objectively measured via ACT and ACQ scores.

## Conclusions

Our findings suggest that dupilumab may offer beneficial effects in managing severely uncontrolled type 2 asthma complicated by tuberculosis. This case report supports the notion that it is feasible to co-administer anti-tuberculosis medications and dupilumab without the need to delay TB treatment. These insights could be valuable for clinical practice and may serve as a foundation for future studies.
